# Visual function and quality of life in patients with Stevens-Johnson syndrome who received acute protocol-based ocular care

**DOI:** 10.3389/ftox.2022.992696

**Published:** 2022-11-02

**Authors:** Swapna S. Shanbhag, Mohammad A. Tahboub, James Chodosh, Hajirah N. Saeed

**Affiliations:** ^1^ The Cornea Institute, L. V. Prasad Eye Institute, Hyderabad, India; ^2^ Department of Ophthalmology, Massachusetts Eye and Ear, Harvard Medical School, Boston, MA, United States; ^3^ Department of Ophthalmology, Illinois Eye and Ear Infirmary, University of Illinois Chicago, Chicago, IL, United States; ^4^ Department of Ophthalmology, Loyola University Medical Center, Chicago, IL, United States

**Keywords:** stevens-johnson syndrome (SJS), toxic epidermal necrolysis (TEN), visual function, national eye institute visual function questionnaire (NEI-VFQ-25), ocular surface disease index (OSDI)

## Abstract

**Purpose:** To report visual function and quality of life (VF/QOL) using the National Eye Institute Visual Function Questionnaire (NEI-VFQ-25) and the ocular surface disease index (OSDI) in patients in the chronic phase of Stevens-Johnson syndrome/toxic epidermal necrolysis (SJS/TEN).

**Methods:** The NEI-VFQ-25 questionnaire was administered to 15 patients who received protocol-based care in the form of topical medications with or without amniotic membrane transplantation (AMT) for acute SJS/TEN. The scores obtained were compared with scores from a healthy population. The associations between the NEI-VFQ-25 and dry eye symptoms as measured by OSDI questionnaire were also studied.

**Results:** Patients were surveyed at a mean of 4.47 ± 2.22 years after acute SJS/TEN. Eleven patients received AMT in the acute phase. The median best corrected visual acuity at the time of administration of the questionnaire was 20/20. The mean composite NEI-VFQ-25 score was 86.48 ± 12. Patients who received protocol-based treatment in the acute phase of SJS/TEN had comparable NEI-VFQ-25 scores with healthy subjects on all subscales except ocular pain (*p* = 0.027) and mental health (*p* = 0.014), which were significantly reduced. The NEI-VFQ-25 composite scores significantly correlated with OSDI (R = -0.75, *p* = 0.001).

**Conclusion:** A protocol-based management strategy composed of early ophthalmic evaluation, grading based on severity, the use of topical corticosteroids and AMT in the acute phase of SJS/TEN in patients with ocular complications helped preserve the VF/QOL. This study highlights the impact of appropriate management of the ocular complications in the acute phase of SJS/TEN.

## Introduction

Stevens-Johnson syndrome/toxic epidermal necrolysis (SJS/TEN) are a spectrum of disease acutely affecting the skin and the mucous membranes (Kohanim et al., 2016). These conditions cause significant necrolysis and desquamation of the skin and mucosae with involvement of the oral, conjunctival, and genital mucosa being most frequent (Shanbhag et al., 2020a). In the acute phase, SJS/TEN affects the ocular surface in the form of denudation of the epithelium of the lid margin, conjunctiva, and cornea (Gregory, 2011). If these complications are not addressed quickly and appropriately, they can progress to long-term sequelae such as lid margin keratinization, symblepharon, dry eye disease (DED), corneal vascularization, and limbal stem cell deficiency (LSCD), which could culminate in bilateral corneal blindness in the chronic phase (Di Pascuale et al., 2005; Gueudry et al., 2009; Basu et al., 2018). Ocular sequelae are reported to be one of the most common and most disabling long-term sequelae in patients with SJS/TEN (Yang et al., 2016; Lee et al., 2017).

For patients who present with ocular involvement in the acute phase of SJS/TEN, a protocol-based management strategy including early ophthalmology consult, grading based on severity of ocular findings, usage of topical corticosteroids, and amniotic membrane transplantation (AMT), when indicated, has been reported to reduce the incidence of vision-threatening complications in the chronic phase (Shanbhag et al., 2019b). Reports on the long-term outcomes of AMT in SJS/TEN have been recently published to show that AMT, when done within the window of opportunity (5–7 days of symptom onset), can mitigate vision-threatening complications in the chronic phase (Shanbhag et al., 2020b; Yang et al., 2020). However, lid-related complications and DED may persist. Outcomes in cases that have been managed in a standardized fashion in the acute phase of SJS/TEN have not yet been reported from the patient’s perspective. The 25-item National Eye Institute Visual Function Questionnaire (NEI VFQ-25) was developed in 1995 to assess visual health and the influence of visual impairment on health-related quality of life (Mangione et al., 2001). This study was undertaken to report the vision-related quality of life (VF/QOL) and the symptoms of ocular surface disease utilizing the NEI-VFQ-25 questionnaire and the ocular surface disease index (OSDI) scoring system, respectively, in patients who received protocol-based management for ocular involvement in the acute phase of SJS/TEN.

## Materials and methods

### Approval

This study was approved by the institutional review board of the Massachusetts Eye and Ear (MEE). The study was conducted under Health Insurance Portability and Accountability Act (HIPAA) compliance and adhered to the tenets of the Declaration of Helsinki.

### Patient selection

**FIGURE 1:**
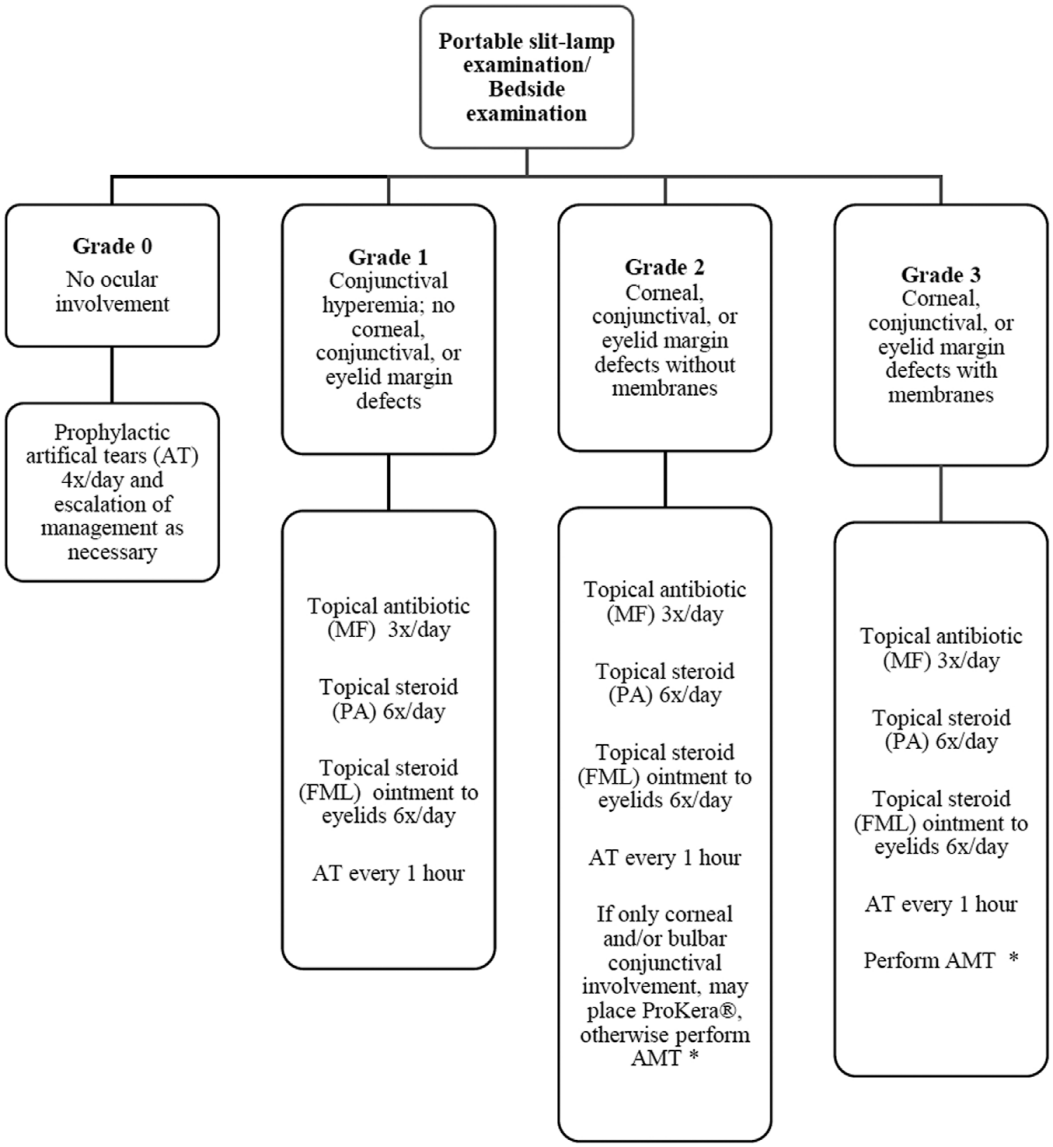
Flow diagram outlining protocol for management of ocular manifestations in acute Stevens-Johnson syndrome/toxic epidermal necrolysis. (MF = moxifloxacin 0.5%; PA = prednisolone acetate 1%; FML = fluorometholone 0.1%; AT = artificial tears; AMT = amniotic membrane transplantation). Decision to perform AMT based on feasibility (intubation status, cooperation, *etc.*). ProKera is acceptable if only bulbar conjunctival or corneal involvement is present or when AMT is not feasible. Reprinted with permission from Shanbhag SS, Rashad R, Chodosh J et al. Long-Term Effect of a Treatment Protocol for Acute Ocular Involvement in Stevens Johnson Syndrome/Toxic Epidermal Necrolysis. Am J Ophthalmol, 208, 331–341.

All patients who received protocol-based management for ocular disease in the acute phase of SJS/TEN and visited the clinic for follow-up between November 2017 and June 2018 were interviewed in this prospective study. This included a subset of SJS/TEN patients who had onset of disease between January 2011 and July 2017. The SJS/TEN protocol that was followed for the care of these patients in the acute phase has been previously described and is shown in Figure 1 (Shanbhag et al., 2019b). Patients who received AMT underwent the procedure as previously described. (Ma et al., 2016; Shanbhag et al., 2019a). Data regarding demographic information, employment status, the etiology of the SJS/TEN, the time interval from AMT to the day the questionnaire was administered, the best corrected visual acuity (BCVA) on the day of the interview, the status of DED, ocular findings of trichiasis and distichiasis, and the history of usage of scleral contact lenses, were collected before the questionnaire was administered. Only patients who spoke English and were above the age of 18 years were interviewed.

### Outcome measures

The NEI VFQ-25 data were administered in an interviewer based-format and collected prospectively from each patient, as described in Version 2000 of the NEI-VFQ-25 ^[12–13]^. The NEI VFQ-25 questionnaire consists of 25 questions with 11 subscales that include questions specific for general health, general vision, ocular pain, difficulty with activities related to distant vision, difficulty with activities related to near vision, limitations in social functioning due to vision, mental health and problems with mental well-being due to vision, role limitations due to vision, dependency due to vision, difficulties with the activity of driving, problems with color vision, and limitations with peripheral vision (Mangione et al., 2001). The NEI-VFQ-25 composite and subscale scores were calculated according to the manual [13]. The primary outcome for this study was the composite NEI-VFQ-25 score. The NEI-VFQ-25 scores range from 0 to 100, with lower scores indicating more ocular symptoms resulting in a poorer quality of life. The mean NEI-VFQ-25 subscale scores from patients who were administered the questionnaire in this study were compared with the mean NEI-VFQ-25 subscale scores of 122 healthy subjects with no ocular disease (except refractive errors with BCVA ≥20/25 in the worse eye) from the original NEI-VFQ-25 study (Mangione et al., 2001). The mean age of the reference population was 59 ± 14 years with all subjects above 21 years. The median visual acuity in the better eye was 20/20.

The OSDI questionnaire was administered to each patient in an interview-based format. This questionnaire consists of three subscales with questions on ocular discomfort, how these symptoms limit activities such as reading, and how environmental triggers can impact dry eye symptoms (Schiffman et al., 2000). Each question is graded in accordance with the frequency of the symptoms, with a score of “0” correlating with symptoms occurring “none of the time” and a score of “4” correlating with symptoms occurring “all the time.” The OSDI scores can range from 0 to 100, with higher scores indicating greater disability due to symptoms related to DED.

### Data and statistical analysis

The statistical analysis was performed using Stata statistical software 15 (StataCorp, College Station, Texas). Normality of the data were evaluated with the Shapiro-Wilk test. Quantitative variables were expressed as mean ± standard deviation (SD), and qualitative variables were expressed as percentages. Visual acuities were measured with a standardized Snellen chart and converted to logMAR values for analysis. Comparison of NEI-VFQ-25 subscale scores with the subscale scores of the reference population was performed with an unpaired *t*-test. The Spearman correlation coefficient was used to test the associations between the NEI-VFQ-25 subscale scores and OSDI scores, between the NEI-VFQ-25 composite scores and OSDI scores, and between the NEI-VFQ-25 composite scores and patient-related parameters such as patient age, BCVA in the worse eye, and the duration of time since the acute phase of SJS/TEN. A two-sided *p* value of <0.05 was considered statistically significant.

## Results

The mean age of the patients to whom the NEI-VFQ-25 questionnaires were administered was 37.3 ± 14 years (range: 20–59). The mean time interval between the acute episode of SJS/TEN and the time at which the questionnaires were administered was 4.47 ± 2.22 years (range: 0.85–9.1). The characteristics of all the patients included in this study are mentioned in Table 1.

**TABLE 1:** Characteristics of patients who underwent protocol-based management in the acute phase of Stevens-Johnson syndrome/toxic epidermal necrolysis.

Characteristics	
Number of patients//eyes	15//30
Gender, Male: Female	3:12
Age, mean ± SD	37.3 ± 14
Interval since acute SJS/TEN in years, mean ± SD	4.47 ± 2.22
Etiology of SJS/TEN	
Drug-induced	
Cotrimoxazole	9
NSAIDs	2
Lamotrigine	1
Others	2
Unknown	1
Employment status	
Employed	12
Student	3
Unemployed	0
Grade of ocular involvement in the acute phase^*^ (median)	2
Number of eyes that received AMT in the acute phase	22
BCVA at the time of administration of questionnaire, Median (IQR)	0 (0–0.1) [LogMAR]
	20/20 (20/20–20/25) [Snellen equivalent]
Number of eyes with dry eye disease in the chronic phase^a^	
Grade 2	6
Grade 3	4
Number of eyes that use scleral lenses in the chronic phase	8
Number of eyes with trichiasis and distichiasis in the chronic phase	12
Number of eyes that underwent additional surgery post AMT	1

SJS/TEN, Stevens-Johnson syndrome/toxic epidermal necrolysis; IQR, Inter-quartile range; SD, standard deviation; BCVA, best corrected visual acuity.

^*^ Grading as per Sotozono classification (Sotozono C, et al.; Japanese Research Committee on Severe Cutaneous Adverse Reaction. Predictive Factors Associated With Acute Ocular Involvement in Stevens-Johnson Syndrome and Toxic Epidermal Necrolysis. Am J Ophthalmol. 2015; 160:228–237. e2. doi: 10.1016/j.ajo. 2015.05.002).

^a^ Grading of dry eye disease as per DEWS, criteria.

The median OSDI score for patients who underwent protocol based-management for acute SJS/TEN was 18.75 (interquartile range: 10.41–45.83). The mean NEI-VFQ-25 composite score was 86.48 ± 12 (range: 56–100). The mean NEI-VFQ-25 composite and subscale scores and the comparison to the scores in the reference population are shown in Table 2. The subscale scores for ocular pain (*p* = 0.027) and mental health (*p* = 0.014) were significantly reduced in the patients with SJS/TEN as compared to the healthy population. The scores were comparable for all other subscales.

**TABLE 2:** 25-item National Eye Institute Visual Function Questionnaire scores in patients who received protocol-based management for ocular manifestations of Stevens-Johnson syndrome/toxic epidermal necrolysis.

NEI-VFQ-25 Scales	Mean ± SD (range) in SJS/TEN patients (n = 15)	Mean ± SD (range) in reference patients (n = 122)	*p* Value
General health	63.33 ± 21 (25–100)	69 ± 24	0.34
General vision	80 ± 13 (60–100)	83 ± 15	0.42
Ocular pain	73.33 ± 26 (25–100)	90 ± 15	0.027
Near activities	88.34 ± 13 (67–100)	92 ± 13	0.32
Distance activities	88.34 ± 16 (42–100)	93 ± 11	0.29
Driving	77.22 ± 27 (42–100)	87 ± 18	0.19
Color vision	100 ± 0 (100)	98 ± 8	0.006
Peripheral vision	98.33 ± 6 (75–100)	97 ± 10	0.46
Vision specific			
Role difficulties	89.16 ± 21 (25–100)	93 ± 13	0.5
Dependency	91.67 ± 18 (42–100)	99 ± 6	0.14
Social functioning	93.33 ± 11 (62–100)	99 ± 3	0.91
Mental health	70.91 ± 29 (6–100)	92 ± 12	0.014
Overall composite score	86.48 ± 12 (56–100)		

SJS/TEN, Stevens-Johnson syndrome/toxic epidermal necrolysis; NEI-VFQ-25, 25-item National Eye Institute Visual Function Questionnaire; SD, standard deviation.

The correlation between the NEI-VFQ-25 scales and OSDI scores is shown in Table 3. The correlation between the NEI-VFQ-25 composite score and OSDI was statistically significant (R = -0.75; *p* = 0.001). The correlation between all the NEI-VFQ-25 subscales and OSDI was statistically significant except for the subscales of driving, color vision, and peripheral vision.

**TABLE 3:** Correlations of NEI-VFQ-25 subscale scores with OSDI scores in patients who received protocol-based management for ocular manifestations of Stevens-Johnson syndrome/toxic epidermal necrolysis.

NEI-VFQ-25 subscales	R Based on OSDI score	*p* Value
General health	-0.6	0.017
General vision	-0.56	0.03
Ocular pain	-0.69	0.004
Near activities	-0.6	0.017
Distance activities	-0.5	0.03
Social functioning	-0.5	0.029
Mental health	-0.8	0.0003
Role difficulties	-0.6	0.02
Dependency	-0.56	0.03
Driving	-0.32	0.2
Color vision	-	-
Peripheral vision	-0.31	0.26
Overall composite score	-0.75	0.001

SJS/TEN, Stevens-Johnson syndrome/toxic epidermal necrolysis; NEI-VFQ-25, 25-item National Eye Institute Visual Function Questionnaire; R = correlation coefficient.

There was no correlation between the composite NEI-VFQ-25 score and the duration of time from acute SJS/TEN (R = 0.09; *p* = 0.73) and the BCVA in the worse eye (R = -0.46; *p* = 0.08). However, there was a statistically significant correlation between the composite NEI-VFQ-25 score and the age of the patient (R = 0.64; *p* = 0.009). There was no correlation between the OSDI score and the duration of time from acute SJS/TEN (R = -0.09; *p* = 0.75), the BCVA in the worse eye (R = 0.36; *p* = 0.19) and the age of the patient (R = -0.4; *p* = 0.14).

## Discussion

SJS/TEN is a life-threatening condition, and survivors of SJS/TEN can suffer from a multitude of complications affecting various organ systems in the chronic phase many years after the initial acute episode (Shanbhag et al., 2020a). Ocular disease in particular is common in SJS/TEN patients and can be debilitating. In one study, 77% of survivors had ocular complications in the chronic phase, and all patients had ocular involvement in the acute phase (Haber et al., 2005). Patients who suffer from chronic ocular complications such as DED and chronic photophobia in the chronic phase of SJS/TEN have significantly lower overall health-related quality of life compared to the normal population (Haber et al., 2005). In a recent questionnaire-based study, out of 57 survivors of SJS/TEN, 70% patients had chronic ocular complications; DED was the most common complication affecting 87% of patients (Ingen−Housz-Oro et al., 2020). Between one-third and two-thirds of patients in this study with chronic ocular complications had difficulty using a computer or cellular phone, watching television, or driving a car. Another study of 17 survivors of SJS/TEN conducted at a mean follow-up of 51.6 months after the acute phase showed that only 29% were employed (Dodiuk−Gad et al., 2016). A significant number of patients require multiple surgical interventions in the chronic phase for ocular complications along with life-long topical medications which contribute to increased financial burden (Ingen−Housz-Oro et al., 2020).

Our study shows that patients who receive an early ophthalmology consult, have acute severity graded based on ocular findings, and are treated with a protocol comprising of topical corticosteroids, topical antibiotics, and AMT have NEI-VFQ-25 scores comparable to the general population for most subscales. We have shown previously that patients treated with this protocol had significantly better visual outcomes and fewer vision-threatening complications over a median follow-up period of 2.6 years after acute SJS/TEN (Shanbhag et al., 2019b).

Previous studies have demonstrated higher OSDI scores that correlate with higher functional impairment and severity of DED in patients with chronic ocular complications post SJS/TEN. In a study by Gueudry et al., at a mean duration of 82 months from discharge for acute SJS/TEN, the median OSDI score for 31 patients with ocular sequelae was 41.6 (range: 0–97.5), corresponding to severe functional impairment (Gueudry et al., 2009). In our study, the OSDI score was 18.75, which corresponds to normal to mild functional impairment due to DED. In the former study, 89% of patients received topical antibiotics, 63% of patients received topical lubricants, only one out of 31 patients received topical corticosteroids, and no patients received AMT in the acute phase of SJS/TEN despite 12 and 13 eyes being described as moderate or severe, respectively. In comparison, in our study, all 30 eyes of 15 patients received topical antibiotics, topical lubricants, and topical steroids, while 22 eyes of 11 patients received AMT in the acute phase. While the baseline patient characteristics were likely different between these two studies, it does not appear that a standardized protocol was followed in the former study. In another study by Tougeron-Brousseau et al., the mean OSDI in 36 patients with chronic SJS/TEN was found to have improved significantly after scleral lens placement (Tougeron−Brousseau et al., 2009). It is unclear what kind of treatment these patients received in the acute phase. Compared to the severe functional impairment demonstrated by the OSDI scores in the study by Tougeron-Brousseau et al., the OSDI score in our study was lower and only four patients in our cohort required scleral lenses in the chronic phase.

Previous studies have also demonstrated that lower NEI-VFQ-25 scores correlate to poorer VF/QOL in patients with chronic ocular complications post SJS/TEN. In the study by Tougeron-Brousseau et al., the mean composite NEI-VFQ-25 score in 32 patients with chronic SJS/TEN was 25.1 ± 16.8 and 67.4 ± 22.1 at presentation and 6 months after scleral lens placement, respectively (Tougeron−Brousseau et al., 2009). Other studies have also shown low NEI-VFQ-25 scores in patients with chronic SJS/TEN. Again, details of acute phase management are not discussed in these studies and it does not appear that standardized treatment protocols were followed. (Kaido et al., 2004) (Papakostas et al., 2015 ^)^. In our study, the mean composite NEI-VFQ-25 score was 86.48 ± 12 in the chronic phase of SJS/TEN, higher than in all previously reported studies on SJS/TEN, and not significantly different than that of a healthy reference population.

In our study, the subscale scores for ocular pain and mental health were significantly reduced in patients with SJS/TEN as compared to the healthy population, despite a median BCVA of 20/20. These findings suggest that it is not just visual acuity that affects vision-related quality of life. It has been shown in various studies that survivors of SJS/TEN have higher incidences of depression, anxiety, and post-traumatic stress disorder and decreased health-related quality of life (Dodiuk−Gad et al., 2016; Hefez et al., 2019; Hoffman et al., 2021). Patients with SJS/TEN, even with good vision, may suffer from ocular discomfort and pain, affecting their daily activities. The etiologies in these specific cases is not known but may be due to DED and coexisting pathologies such as trichiasis and distichiasis (Kaido et al., 2006). Color vision was also found to be significantly different between the two groups, with the reference population scoring worse. We believe this is due to several factors. The question specific to color vision on the VFQ is “Because of your eyesight, how much difficulty do you have picking out and matching your own clothes?“. The answer to this question may reflect mobility issues in addition to color vision. Combined with the older age of the otherwise healthy reference population vs. our SJS/TEN population, this result is likely confounded by age. Additionally, the difference between a mean score of 100 vs. 98 is not clinically significant.

The most common etiology for SJS/TEN in our study was sulfonamide drugs, specifically cotrimoxazole. A recent study demonstrated that patients with SJS/TEN secondary to cotrimoxazole had significantly less severe chronic stage ocular complications and better long-term visual acuity as compared to patients who had SJS/TEN secondary to lamotrigine (Rashad et al., 2021). This could be a contributing factor to the better NEI-VFQ-25 OSDI scores in our cohort, thus reflecting a better VF/QOL and lesser severity of DED. However, we believe that a more significant contribution to the NEI-VFQ-25 and OSDI scores in our cohort is that all patients received an early ophthalmology consult after the diagnosis of SJS/TEN, received timely and appropriate protocol-based treatment, and were asked to follow-up frequently in the chronic phase. This ensured that any issues in the acute, sub-acute, and chronic phases were addressed expediently.

This study highlights the role of timely and appropriate acute care for ocular involvement in patients with SJS/TEN. Recent studies have shown that only 66% of burn centers in the United States routinely get an ophthalmology consult for their SJS/TEN patients (Le et al., 2016). Another study noted that although 67% of their patients admitted for SJS/TEN had ophthalmologic complications in the acute phase, only 6% were followed by an ophthalmologist in the chronic phase after discharge (Olteanu et al., 2018). We believe that if every patient with SJS/TEN receives an ophthalmology consult in the acute phase, is graded for severity, treated and managed in a standardized and appropriate fashion, and has the necessary follow-up in the chronic phase for any new potential issues, the VF/QOL of these patients can be optimized. Indeed, we have previously shown that a standardized protocol-based approach to acute ocular care can reduce chronic complications, and it follows that VF/QOL would also be improved. Barriers to implementing such care may include the time and coordination that an AMT procedure can take. However, we have previously reported a technique which significantly reduces the time and resources necessary for the procedure (Shanbhag 2019a).

The limitations of this study include its small sample size and the absence of the NEI-VFQ-25 and OSDI scores in a control group that did not receive protocol-based care in the acute phase of SJS/TEN. However, patients who did not receive protocol-based care in the acute phase of the disease were often patients who received no or poor acute care and went on to undergo surgeries such as keratoprosthesis that would confound the NEI-VFQ-25 scores. Another limitation is that all patients seen in the acute phase were not seen in the chronic phase, resulting in selection bias. However, it is likely that those who did follow up had more severe disease and so the OSDI and NEI-VFQ-25 scores may actually be skewed towards worse symptomatology. Despite this, the scores on both were better than those reported in previous studies. Lastly, Schirmer’s test scores may have been a useful adjunct in assessing dry eye and, if available, could have been correlated with OSDI and NEI-VFQ25 scores; however, Schirmer’s scores were not consistently recorded and were not analyzed in this study.

SJS/TEN is a rare condition, and patient-reported outcomes several years after acute SJS/TEN, as reported in this study, add to our knowledge about the impact that treatment in the acute phase can have on the quality of life of these patients. Hence, we conclude that efforts should be directed at spreading awareness among physicians about the need for an ophthalmology consult in the acute phase immediately after SJS/TEN is suspected, and emphasizing the need to follow an evidence-based treatment protocol as we have described.

## Data Availability

The raw data supporting the conclusion of this article will be made available by the authors, without undue reservation.
